# Influence of Donor Thickness on Visual Acuity in Descemet’s Stripping Automated Endothelial Keratoplasty

**DOI:** 10.18502/jovr.v17i4.12296

**Published:** 2022-11-29

**Authors:** Tomislav Kuzman, Ana Meter, Miro Kalauz, Sanja Masnec, Ivan Škegro, Ivana Jonjić

**Affiliations:** ^1^Department of Ophthalmology, University Hospital Center Zagreb, Zagreb, Croatia; ^3^https://orcid.org/0000-0002-7137-5059; ^4^https://orcid.org/0000-0003-1740-9198

**Keywords:** Cornea, Corneal Transplantation, Descemet Stripping Endothelial Keratoplasty, Fuchs's Endothelial Dystrophy

## Abstract

**Purpose:**

Conventional Descemet's Stripping Automated Endothelial Keratoplasty (DSAEK) is a corneal transplantation procedure where the patient's inner dysfunctional layer is replaced with donor lamella. The data currently present in the literature about the correlation between lamellar thickness and visual acuity is sometimes contradictory and lacks clarity.

**Methods:**

Study included 55 eyes that underwent the conventional DSAEK procedure. Patients had no other comorbidities that could affect visual acuity. Data about lamellar thickness and visual acuity were measured six months after surgery with anterior segment optical coherent tomography (A5-OCT).

**Results:**

The results show that visual acuity before surgery improved from 0.82 to 0.25 logMAR after surgery. Better visual acuity of 0.20 logMAR was achieved with postoperative lamellas thinner than 124 μm, while statistically significantly lower visual acuity of 0.29 logMAR was gained with postoperative lamellas thicker than 124 μm.

**Conclusion:**

Our results suggest that the goal after conventional DSAEK is to have postoperative lamellas thinner than 124 μm in the eye, as this will result in better postoperative visual acuity. This value represents the optimal thickness for conventional DSAEK surgery that could minimize tissue loss for eye banks and surgeons may experience fewer problems during surgery, while obtaining good final visual acuity.

##  INTRODUCTION

The first contact with the outside world through light transition begins in the cornea which also acts as the first barrier against infection. Thus, millions of people around the world who suffer from corneal scarring may experience total or partial vision loss if remain untreated.^[1]^

Cornea is a multilayered organ composed of the epithelium, stroma, and endothelium. The corneal epithelium is the most anterior nonkeratinized, five to six cells thick layer.^[1]^ The stroma is resilient, collagenous tissue arranged in sheets of fibrils called lamellae. The corneal endothelium is the last but not the least barrier comprising one layer of the cells incapable to regenerate after damage.

The most common diseases causing endothelial damage are Fuchs's endothelial dystrophy and pseudophakic bullous keratopathy. Fuchs's endothelial dystrophy is a slowly progressive disease that usually affects both eyes; more common in women in later life. The key pathophysiological moment is the decay of the endothelial cells that causes increased accumulation of fluid and subsequently edematous stroma leading to cloudy cornea.

The pathophysiological mechanism responsible for bullous keratopathy is the inability of the corneal endothelium to maintain a normally dehydrated cornea. The corneal endothelium trauma can occur after cataract surgery, excessive flow of fluid during surgery, high dissipated ultrasound energy during emulsification of hard cataracts or after applying intracameral agents.^[2,3,4]^ The term pseudophakic bullous keratopathy is used in the occurrence of bullous keratopathy in patients who have had intraocular lens insertion after phacoemulsification (PHACO) surgery. The innovative improvements in corneal transplant surgery has made it possible today to replace the diseased layers of the recipient cornea.

Lamellar endothelial keratoplasty has replaced penetrating keratoplasty (PK) as the standard for treating endothelial corneal diseases. The most common surgical technique is Descemet stripping automated endothelial keratoplasty (DSEAK) which has rapidly become the preferred alternative to PK because of its smaller incision size, faster rehabilitation, and minimal induction of astigmatism and refractive shift.^[5,6,7]^ DSAEK employs mechanical stripping of the host endothelium and replaces it with a healthy homograft of posterior corneal lenticule harvested using an automated microkeratome.^[6]^ Advantage of DSAEK over PK includes preservation of biomechanical corneal properties because there is no need for corneal stitches.^[8]^

Further development in DSAEK graft preparation encouraged toward more delicate and thinner lamellas, so surgeons could use nano-thin ( < 50 μm), ultra-thin ( < 100 μm), or conventional ( > 100 μm) grafts.^[9]^ Surgeons' special requests to eye banks for thin and ultra-thin graft thickness have led to real implications including: increased risks of tissue loss during graft processing, and during the logistics of tissue distribution with increased associated costs and pricing for special tissue processing.^[10]^ Over time, there has been a change in the definition of subtypes of the DSAEK method depending on the thickness of the lamella. As confirmed by a recent study, the conventional and ultra-thin DSAEK methods' definitions have been confirmed by 56% of experienced surgeons who defined donor lamellar thickness < 100 μm as ultra-thin DSAEK and lamellar thickness > 100 μm as the conventional DSAEK method. Conventional DSAEK is the most used keratoplasty procedure in the United States.^[11]^

The data currently present in the literature about the correlation between lamellar thickness and the visual acuity is sometimes contradictory and lacks clarity [Table 1]. Many authors found that there is correlation between final visual acuity and thickness of donor lamella, concluding that transplanting thinner donor lamellas result in better visual acuity.^[6,8,10,12,13,14]^ On the other hand, there are also many authors whose results found no correlation between postoperative lamellar thickness and visual acuity after DSEAK surgery, suggesting that donor graft thickness did not influence final visual acuity.^[15,16,17,18,19,20]^ Some authors, for example, Tourabaly et al in recent papers suggest that there were no significant differences in final visual acuity between all investigated groups with varied postoperative lamellar thickness which includes lamellas from nano-thin grafts (15–49 μm) all through to very thick group of grafts (150–250 μm).^[20]^ Such significant inconsistencies inspired this research. This research would encourage more accurate and explicit definitions of prospective criteria when selecting the conventional DSEAK method. In addition, when performing DSEAK operations, more consideration would be paid toward frame design and focusing on establishing clearer lamellae thickness criteria which would encourage improved tissue safety, technical concerns, and result in better surgical outcomes such as enhanced visual acuity improvement. However, preparation and handling of thinner grafts would not be without challenges;^[8]^ such as determining whether the surgeons in an effort to easily perform surgical procedures while achieving optimal visual outcomes for their patients should insist on creating thinner graft tissue, or determining how thin the donor lamellas should be in order to avoid tissue loss during preparation.

**Table 1:** Influence of lamella thickness on visual acuity with data regarding to the author, number of patients, method of measurement, and lamellar thickness.


**Author**	***N***	**Method**	**Avg lamella preop (µm)**	**Avg lamella postop (µm)**	**Influence of lamella thickness on visual acuity**
Pogorelov et al^[6]^	15	Postop slit-lamp, AS OCT	191	100	Yes
Maier et al^[10]^	65	AS OCT	169	153	Yes
Neff et al^[12]^	33	AS OCT	/	131	Yes
Dickmann et al^[8]^	79	AS OCT	/	97	Yes
Busin et al^[13]^	279	AS OCT Intraop	/	78	Yes
Roberts et al^[14]^	130	Preop US Post AS OCT	95	90	Yes
Cleyenenbreugel et al^[19]^	37	Preop US	175	/	No
Woodward et al^[16]^	64	Post AS OCT	199	165	No
Terry et al^[15]^	418	Preop US Post AS OCT	162	/	No
Phillips et al^[17]^	144	Preop US Post AS OCT	145	/	No
Ivarsen et al^[18]^	125	Post AS OCT	/	149 (*n* = 11) 183 (*n* = 19)	No
Tourabaly et al^[20]^	150	Post AS OCT	/	74	No

*N*, number of patients; US, ultrasound pachymetry; AS OCT, anterior segment optical coherence tomography; Avg lamella preop, average lamellar thickness preoperative; Avg lamella postop, average lamellar thickness postoperative					

**Table 2:** Arithmetic mean and associated standard deviation, median and total range of at least to the highest value for variables of best-corrected visual acuity (BCVA) before and after surgery (six months follow-up) *P < 0.05.


	**Surgery**	**M (SD)**	**C**	**TR (Min–Max)**
BCVA	Before	0.15 (0.107) (0.82 logMAR)	0.10*	0.50 (0.10–0.60)
	After six months follow-up	0.57 (0.202) (0.25 logMAR)	0.60*	0.75 (0.10–0.60)

BCVA, best-corrected visual acuity; M, arithmetic mean; SD, standard deviation; C, central value; TR, total range; *P < 0.05				

**Table 3:** *T*-test for differentiated lamellar thickness between preoperative and postoperative determined according to the lamellar thickness.


	**Lamellar thickness (µm)**	***N***	**M (SD)**	***t*** **-test**
BCVA	≤ 124	28 (50.9%)	0.63 (0.162) (0.2 logMAR)	
	> 124	27 (49.1%)	0.51 (0.221) (0.29 logMAR)	2.390*
*P * < 0.01		

*t*-test, test for paired samples; M, arithmetic mean; SD, standard deviation; BCVA, best-corrected visual acuity				

##  METHODS

The study was approved by the Ethics Committee of University Hospital Centre Zagreb (approved 19.09.2017.; Reference number: 02/21 AG). All patients signed the informed consent form to take part in the study. The study was performed according to the ICMJE recommendations for protection of research participants and adhering to the newest revision of the Helsinki Declaration.

### Subjects 

This prospective case series study enrolled 55 eyes of patients. Criteria for eligibility were Fuchs endothelial dystrophy (26 patients) and pseudophakic bullous keratopathy (29 patients), that underwent DSAEK. The average patient age was 70.9 +/– 9.4 years. The study excluded patients with other illnesses that might have affected visual acuity, such as macular degeneration, macular edema, diabetic retinopathy, visual nerve damage, postoperative complications and glaucoma. Eyes with preoperative lamellar thickness of < 100 μm were also not considered for the study because tissue processing for ultra-thin or nano-thin DSAEK is not performed in our eye bank as it requires a more intricate procedure which is different from what is utilized in conventional standard DSAEK procedure.

Patient follow-up was six months with the 100% enrollment all through the study. Informed consents were obtained from all patients enrolled in the study.

Each patient underwent a complete ophthalmology examination preoperatively and at six months post treatment which included: best-corrected visual acuity (BCVA; Early Treatment Diabetic Retinopathy Study [ETDRS] chart – logMAR table), Goldmann aplanation tonometry, Slit-lamp examination and fundus evaluation. Information about the thickness of cornea and lamella were examined by anterior segment OCT Visante (Carl Zeiss Meditec, Jena, Germany).

The corneas were stored in the University Hospital Centre Zagreb Eye Bank in either hypothermic storage or tissue culture media. Corneas in a hypothermic storage medium were stored at a temperature of 4ºC for a maximum of seven days, and were then prepared for lamellar keratoplasty immediately before planned operation. The other corneas were kept in a tissue culture media at a temperature of 31ºC for a maximum of 28 days. During storage in tissue culture, the media was microbiologically controlled. At the end of storage, the endothelial cell viability and morphology were determined. Corneas during storage in tissue culture becomes much thicker.^[21]^ To return to the physiological thickness, it must be stored in a transport media containing dextran, for at least for 24 hr before the preparation for lamellar keratoplasty. Due to the short corneal validity in the transport media (depending on the manufacturer, Alchemy five days, Eurobio four days), the cornea were placed in the transport medium only when assigned to patient. The transport medium was kept until the third microbiological control that was taken in the incubator at 31ºC. After taking the last microbiological control, it was kept in the thermostat at 22ºC until release. The procedure for preparing corneas for lamellar keratoplasty was performed by specially trained employees using the automatic microkeratome (Gebauer Slc Original, Neuhausen, Germany). Each cornea was placed on the artificial eye chamber filled with corneal storage media to preserve the endothelial cell viability. The epithelium of each cornea was removed to make the cut as precise as possible. The permissible variation of the knife, which was 30 μm, should be considered. The cornea was carefully removed from the artificial eye chamber and stored in a transport media for delivery to transplantation center.

All patients underwent DSAEK and were operated in University Hospital Centre Zagreb, Croatia. The DSAEK was performed by one surgeon using a standardized operative technique. All surgeries were performed under general anesthesia. The patients with Fuchs dystrophy and cataract underwent combined phacoemulsification and DSAEK surgery. Descemet stripping was performed under air bubble or with use of viscoelastic during combined procedures. Implantation of lamella was performed with use of Busin glider to minimize tissue trauma. The lamella was attached with air bubble.

### Statistics 

Microsoft SPSS statistical package for Windows was used. Results were expressed as mean value and standard deviation (SD). Differences between the groups were tested by Student's *t*-test. A *P*-value of < 0.05 was considered statistically significant.

##  RESULTS

The current study included a total of 55 eyes of 55 patients (34 female, 61.8%) aged 48 to 73 years. The average age was 70.9 years.

Table 2 shows the data of descriptive statistics (arithmetic mean and associated standard deviation, median and total range of at least to the highest value) for variables of visual acuity before and six months after surgery. The preoperative BCVA ranged from a minimum of 1.00 logMAR to a maximum of 0.22 logMAR and postoperatively from 1.00 logMAR to 0.07 logMAR. The average BCVA before surgery was 0.82 logMAR while after surgery improved to 0.25 logMAR which was statistically significant (*P* < 0.05 *t*-test for pair samples).

Subjects were divided in two groups: above or under the donor lamella thickness median after surgery (C = 124 μm) in order to determine whether the visual acuity was statistically significant to the lamellar thickness. We observe them as a group of 50% of the subjects below and the group of 50% of the subjects above the central value (C = 124 μm). Table 3 shows 28 subjects (50.9%) had a lamella thickness of 124 μm or less, while for the remaining 27 subjects the lamella was above 124 μm. There was a statistically significant difference in the mean values of these two groups (*t* = 2.390 *P* < 0.01). Better visual acuity of 0.20 logMAR was achieved with lamellas thinner than 124 μm, while statistically significant lower visual acuity was 0.29 logMAR for lamellas thicker than 124 μm.

##  DISCUSSION

The results of our study show that in conventional DSAEK, donor lamella thickness correlates with the BCVA, suggesting that thinner lamellae may result in better visual acuity. All factors related to visual acuity improvement are being intensively investigated as endothelial keratoplasty has become more widely used today than full corneal PK when treating corneal endothelial disease.^[22,23]^ DSAEK, unlike PK, provides faster recovery, less postoperative eye irritation, less postoperative refractive error, better visual acuity, and less invasive eye surgery.^[24]^ The method of transplanting only the Descemet membrane called DMEK (Descemet membrane endothelial keratoplasty) was introduced as an improvement to the technique of endothelial keratoplasty. In DMEK, only Descemet's endothelial membrane without adjacent stroma is transplanted. DMEK gives superior results of postoperative visual acuity compared to DSAEK or PK.^[25,26]^ Through experience, it became clear that the thin part of the stroma transplanted in DSAEK affects the poorer visual acuity compared to DMEK. Unlike DSAEK, DMEK although a better method in terms of final visual acuity is not still the most popular due to the long learning curve and the complexity of the operative technique.^[25,27,28]^ As DSAEK became the method of choice for the treatment of endothelial diseases of majority of surgeons, they tried to reduce the thickness of the lamella to catch up with the visual acuity results achieved with DMEK. Although many methods have been developed to prepare extremely thin lamellas in a nonstandard way, they have not yet entered into wider application. Further developments in the preparation of lamellas for DSAEK successfully created thinner lamellas, resulting in lamellas characterized as nano-thin lamellae thinner than ≤ 50 μm, then ultra-thin lamellae thinner than ≤ 100 μm, and conventional lamellae thicker than ≥ 100 μm.^[9]^ In addition to the special techniques required in preparing ultra-thin lamellae, there are increased risks of tissue loss in tissue processing, logistics, and distribution with increased tissue processing costs.^[10]^ Our research dealt with the lamellae that define the conventional DSAEK method, which is implemented by most eye banks in the world. However, even conventionally prepared lamellas can vary in their thickness from 100 to 250 μm. Although it is much safer for eye bank staff to cut a thicker lamella because the possibility of damage to the transplant is less and although it is easier for the surgeon to manipulate the thicker lamella during surgery, it is still debatable as to whether transplanting thicker donor lamellas results in the same visual acuity as the thin ones.

Studying the literature, we came across several researchers who tried to determine whether there was any correlation between postoperative visual acuity and the thickness of the lamellae used to perform the keratoplasty procedures [Table 1]. After processing our results, we concluded that with conventional DSAEK, surgeons should strive toward selecting lamellae thinner than 124 μm because better visual acuity is achieved, potential tissue loss during eye bank preparation is not influenced, and is sufficient for safe manipulation during surgery. In our study the improvement of visual acuity was from an average of 0.82 logMAR before surgery to 0.25 logMAR after surgery [Table 2]. However, patients with thinner lamellae below 124 µm achieved better visual acuity of 0.20 logMAR than those with lamellae thicker than 124 µm with an acuity of 0.29 logMAR [Table 3]. The average postoperative visual acuity after DSAEK in similar studies generally ranged from 0.45 to 0.15 logMAR and corroborates with our results confirming the success of the procedure itself.^[29]^

Our results have been deemed comparable with other studies that support the thesis that thinner lamellae selected for DSAEK procedures are better for achieving improved visual acuity. Our conclusions were similar with Pogorelov's research which included 15 respondents with deturgescence of donor lamellas from 191 to 100 μm six months after surgery.^[6]^ Deturgescence of donor lamella begins after implantation in the eye and continues for several months. Postoperative thickness of lamella stabilizes six months after surgery, which is why we performed final measurements at that time. Although the study of Maier et al was retrospective, almost identical results were obtained when compared with our study. Their study was conducted on 65 eyes and showed that lamellar thickness of < 120 μm correlated with better visual acuity.^[10]^ Neff et al also obtained similar results in their retrospective study on 33 eyes, and concluded that donor lamellas below 131 μm resulted in better visual acuity.^[12]^ Dekaris et al conducted a similar study and concluded in a 20 subjects that lamellae thinner than 180 μm facilitates better and faster visual recovery than thicker grafts, although measurements were made on the first postoperative day and postoperative deturgescence was not taken into account.^[30]^

There are various studies that suggest that where thinner donor lamellas, in most cases ultra-thin lamellas, were utilized in performing keratoplasty procedures the resultant visual acuity was better as compared to those that utilized thicker lamellas. Although comparative research studies focused on using ultra-thin lamellas, it still supports our thesis even though alternative operative techniques were used. Busin conducted research in 108 eyes and concluded that visual acuity after UT-DSAEK was comparable to DMEK but better than conventional DSAEK.^[13]^ This research is interesting because it showed that visual acuity was better after the application of thin lamellae. However, this research is related to ultra-thin lamellae prepared by a special technique of double-lamella cutting, which is currently not performed in every eye bank. A similar study was conducted by Roberts et al who developed a special technique for preparing ultra-thin lamellae by using the donor tissue dehydration method. This prospective study of ultra-thin lamellae on a group of 130 eyes concluded that thinner donor lamellae transplantation achieve better visual acuity that is close to the results of DMEK transplantation.^[14]^

Cleynenbreugel et al measured lamella thickness in 37 eyes by ultrasound pachymetry intraoperatively and concluded that lamella thickness had no direct effect on visual acuity or endothelial cell count.^[19]^ This study does not take into account postoperative lamella deturgescence.^[31]^ Woodward et al performed a retrospective review on 64 eyes and concluded that there was a poor correlation of BCVA at the final visit with preoperative or postoperative lamella thickness.^[16]^ Possible reasons for this conclusion can be found in the large postoperative range of lamellae from 88 to 335 μm, and various postoperative thickness measurement time. Terry et al conducted a retrospective study on 418 eyes and obtained similar results to our study for a group of lamellae with a range of 80 to 124 μm that had better visual acuity, but eventually concluded that in a large group of lamellae between 100 and 200 μm donor lamella thickness has a weak association with postoperative visual acuity.^[15]^ The mentioned research was limited as preoperative thickness was measured without taking deturgescence into account. A study conducted by Phillips et al on 144 eyes found no correlation between lamella thickness and visual acuity, but the authors compared preoperative thickness of the lamella with postoperative visual acuity.^[17]^ Tourabaly et al in recent papers suggest that there were no significant differences in final visual acuity regardless of lamellar thickness from 15 to 250 μm.^[20]^

It can be concluded that there are studies that confirm our research, while those that reflect the opposite are usually comparing preoperative lamella thickness with postoperative visual acuity. These studies that don't support our research also do not take into account postoperative deturgescence which may contribute toward thinning of the lamella in the eye which stabilizes from four to six months after surgery.^[6,32]^

Some researchers are also focusing on alternative factors that could affect visual acuity in DSAEK. Ivarsen et al investigated the influence of light scattering and total corneal thickness of the recipient on the visual outcome after DSAEK. This study conducted on 125 eyes focused more on corneal densitometry after transplantation, and the authors believe that changes in corneal structure and potential unwanted light scattering at the donor graft and cornea recipient interface are more important for final vision quality than lamella thickness alone.^[18]^ Khakshour et al concluded in a group of 16 eyes that there was a significant correlation between lamellae and corneal interface reflection and visual acuity, emphasizing that higher order corneal aberrations have negative influence on visual acuity.^[33]^ Graffi et al evaluated the relationship between graft thickness, regularity, and visual acuity in 89 eyes and concluded that DSAEK grafts thinner than 100 μm are also more regular than thicker ones, and that in Fuchs endothelial dystrophy thinner grafts result in better visual acuity.^[34]^

Further research is recommended in determining which qualities of the lamellae, that is, either the thickness of the lamellae and their respective architecture or the interface with the recipient's cornea are responsible for better visual acuity postoperatively. Our results suggest that the goal after conventional DSAEK is to have postoperative lamellas thinner than 124 μm in the eye, as this will result in better postoperative visual acuity. This value can be the optimal thickness for conventional DSAEK surgery that could minimize tissue loss for eye banks and the surgeons could have fewer problems during surgery, while obtaining good final visual acuity.

### Ethical Considerations

This study was approved by the Ethics Committee of University Hospital Centre Zagreb (approved 19.09.2017.; Reference number: 02/21 AG). All patients signed the informed consent form to participate in the study.

### Financial Support and Sponsorship

None.

### Conflicts of Interest

There are no conflicts of interest.
